# *Chlamydomonas* POLQ is necessary for CRISPR/Cas9-mediated gene targeting

**DOI:** 10.1093/g3journal/jkab114

**Published:** 2021-04-09

**Authors:** Irina Sizova, Simon Kelterborn, Valeriy Verbenko, Suneel Kateriya, Peter Hegemann

**Affiliations:** 1 Experimental Biophysics, Institute of Biology, Humboldt University of Berlin, Berlin D-10099, Germany; 2 Petersburg Nuclear Physics Institute named by B.P. Konstantinov of National Research Centre “Kurchatov Institute”, Gatchina 188300, Russia; 3 Kurchatov Genome Center - PNPI, Gatchina 188300, Russia; 4 Laboratory of Optobiology School of Biotechnology, Jawaharlal Nehru University, New Delhi 110067, India

**Keywords:** *Chlamydomonas reinhardtii*, CRISPR Cas-induced gene targeting, canonical nonhomologous end-joining (cNHEJ), DNA polymerase theta (POLQ)-dependent microhomology-mediated end joining, KU80, KU70, POLQ, algal biotechnology

## Abstract

The use of CRISPR/Cas endonucleases has revolutionized gene editing techniques for research on *Chlamydomonas reinhardtii*. To better utilize the CRISPR/Cas system, it is essential to develop a more comprehensive understanding of the DNA repair pathways involved in genome editing. In this study, we have analyzed contributions from canonical KU80/KU70-dependent nonhomologous end-joining (cNHEJ) and DNA polymerase theta (POLQ)-mediated end joining on SpCas9-mediated untemplated mutagenesis and homology-directed repair (HDR)/gene inactivation in *Chlamydomonas*. Using CRISPR/SpCas9 technology, we generated DNA repair-defective mutants *ku80, ku70, polQ* for gene targeting experiments. Our results show that untemplated repair of SpCas9-induced double strand breaks results in mutation spectra consistent with an involvement of both KU80/KU70 and POLQ. In addition, the inactivation of *POLQ* was found to negatively affect HDR of the inactivated paromomycin-resistant mut-*aphVIII* gene when donor single-stranded oligos were used. Nevertheless, mut-*aphVIII* was still repaired by homologous recombination in these mutants. *POLQ* inactivation suppressed random integration of transgenes co-transformed with the donor ssDNA. KU80 deficiency did not affect these events but instead was surprisingly found to stimulate HDR/gene inactivation. Our data suggest that in *Chlamydomonas*, POLQ is the main contributor to CRISPR/Cas-induced HDR and random integration of transgenes, whereas KU80/KU70 potentially plays a secondary role. We expect our results will lead to improvement of genome editing in *C. reinhardtii* and can be used for future development of algal biotechnology.

## Introduction

*Chlamydomonas reinhardtii* is a model algae used for study of photoreceptors, photomovement, photosynthesis, various aspects of metabolism and biosynthetic pathways, chloroplast gene expression, and others, as well as for many applications in biotechnology (Salome and Merchant 2019). Improvements in *Chlamydomonas* gene editing have been achieved through the use of the CRISPR/Cas endonuclease system (Ghribi *et al.* 2020). Null mutants for genes with selectable phenotypes were obtained with DNA template-free gene targeting (Jiang *et al.* 2014; Baek *et al.* 2016; Shin *et al.* 2016; Jiang and Weeks 2017; Jeong *et al.* 2018; Guzmán-Zapata *et al.* 2019; Dhokane *et al.* 2020). For genes without a selectable phenotype, direct integration of antibiotic resistance cassettes into the SpCas9 cleavage site allowed editing efficiencies of up to 87% of preselected clones (Shin *et al.* 2016; Shamoto *et al.* 2018; Findinier *et al.* 2019; Kim *et al.* 2020; Picariello *et al.* 2020). Inclusion of 25–50 bp homology arms and performing gene targeting at the G2/M stage of the cell cycle also increased the efficiency of homologous directed gene knockout (Angstenberger *et al.* 2020; Picariello *et al.* 2020). Modifications, such as precise FLAG-tag insertions or nucleotide exchanges using gene-specific single-stranded oligodeoxynucleotides (ssODNs) are also possible (Greiner *et al.*, 2017; Sizova, unpublished data). The efficiency of targeted gene inactivation reached 10–15% for pre-selected clones, but precise homology-directed repair (HDR) outcomes were detected only in 1–2% pre-selected cells. To utilize the full power of CRISPR/Cas system and improve error-free HDR rates in *Chlamydomonas*, it is essential to establish a better understanding of involved DNA repair pathways.

CRISPR/Cas-induced mutations can be the result of error-prone cNHEJ, alternative end joining that includes DNA polymerase POLQ-dependent microhomology-mediated end joining (TMEJ), and HDR (Black *et al.* 2016; Brambati *et al.* 2020). However, selection of the repair pathway depends on many factors, including the phase of the cell cycle, the type of the DNA cut, chromatin structure, chromatin composition, and concentration of the repair template (Boel *et al.* 2018). The cNHEJ and TMEJ pathways are both error-prone and can lead to pathway-specific short nucleotide insertions or deletions, which can inactivate the target gene. Typical cNHEJ mutations are small base pair insertions/deletions occurring at a DSB site. Unlike NHEJ, TMEJ-specific deletions usually occur between short microhomologies on either side of the DSB. TMEJ specific insertions, also termed ‘templated insertions’, are usually 3–30 bp sequences at the sites of DSB joining that result from the synthesis initiated from the free 3′ end of a resected DSB end. This resected 3′ end anneals to a short complementary sequence inside a DSB flanking region in either *cis, trans*, or sometimes to sequences many kb apart. The 3′ end can also anneal to sequences located on another chromosome (Black *et al.* 2016; Wyatt *et al.* 2016; Schimmel *et al.* 2017; Carvajal-Garcia *et al.* 2020). However, microhomology is not necessarily essential for end-joining, and TMEJ can function in either the absence of microhomology or in the presence of a single 3′ terminal base pair of microhomology (Black *et al.* 2016; Kent *et al.* 2016).

If an exogenous homologous DNA repair template has been supplied, a CRISPR/Cas-mediated DSB can be precisely modified through HDR. HDR is a complicated process with three sub-pathways: (1) double-strand break repair (DSBR) through the formation of Holliday junction structures, (2) synthesis-dependent strand annealing (SDSA), and (3) single-strand DNA incorporation (ssDI; Kan *et al.* 2017). Earlier studies in mammalian cells have demonstrated that the Rad51-dependent DSBR pathway was the predominant mode of precise gene insertion if the plasmid donors used contained long (>500 bases) homology arms flanking long central heterologies (Yang *et* al. 2013; Kan *et al.* 2014; Danner *et al.* 2017). However linear fragments with short homologous arms (5–48 bp), can be efficiently and precisely integrated into the DSB via the SDSA pathway without the template being inserted at the DSB site (Nakade *et al.* 2014; Hisano *et al.* 2015; Paix *et al.* 2017; Yao *et al.* 2017; Wierson *et al.* 2020; Huang and Puchta 2019). ssODN templates utilize either the SDSA or ssDI pathway, depending on the type of a genomic lesion (Kan *et al.* 2017; Kan and Hendrickson 2019). In the presence of either DSBs or paired nicks, the SDSA pathway has been reported to be preferentially utilized (Paix *et al.* 2017; Kan *et al.* 2017; Boel *et al.* 2018). Complex genomic insertions of the donor templates often found in SDSA products are hypothesized to result from repeated ‘template switching’ (Boel *et al.* 2018) when a newly synthesized strand anneals to the second template and continues DNA synthesis by combining sequences from partially overlapping DNA fragments. The ssDI pathway, in which a ssODN is physically incorporated into a single-strand gap and displaces the flanking sequences on both sides, has been described for repair of single strand breaks (Kan *et al.* 2017).

The functional role of POLQ in DSB repair has been analyzed in nematodes (van Schendel *et al.* 2015), fish (Thyme and Schier 2016), plants (van Kregten *et al.* 2016), mammals (Mateos-Gomez *et al.* 2015), and moss *Physcomitrella* (Mara *et al.* 2019). However, its homologs have not been found in either yeast or fungi (Brambati *et al.* 2020). Microalgae like *C. reinhardtii* contain a putative *POLQ* homolog gene (in *C. reinhardtii*, Cre16.g664300), but this gene’s role in DSB repair and gene targeting is unknown. The DNA polymerase POLQ is a unique eukaryotic polymerase. It possesses both an A-family DNA polymerase domain and an SF2 helicase-like domain, which likely assists in microhomology-mediated end joining and suppresses homologous recombination (HR) via its anti-recombinase activity (Black *et al.* 2016; Wood and Doublié 2016; Wyatt *et al.* 2016; Brambati *et al.* 2020).

In this study, we analyzed the contribution of the cNHEJ and TMEJ for the repair of CRISPR/Cas-induced DSB in *Chlamydomonas*. We generated mutants deficient in KU70, KU80, and POLQ in wild type (wt) cells and a strain containing a defective antibiotic resistance gene and then used them for gene targeting experiments. We found that CRISPR/Cas-induced DSBs can be repaired by HR, NHEJ, or TMEJ, with TMEJ being the dominant pathway for gene targeting. Template-free repair of SpCas9-induced DSBs results in mutation spectra suggesting that both KU80/KU70 and POLQ are responsible for gene modification. POLQ is the main contributor for HDR and random integration of transgenes with CRISPR/Cas, whereas KU70/KU80 appears to be less important. Loss of POLQ caused a sharp drop in gene targeting efficiency. However, the loss of KU80 stimulated gene targeting with ssODN repair templates. Linear DNA fragments, including short gene-specific homology arms, could potentially serve as the best donors for efficient CRISPR/Cas-mediated gene targeting in *Chlamydomonas*. We believe that by optimizing the homology arm length and the concentration of the DNA repair template, the efficiency and accuracy of gene editing can be improved. Therefore, our findings and results open promising possibilities for improving gene targeting and editing in *Chlamydomonas*.

## Materials and methods

### Strain and culture conditions

Motile *C. reinhardtii* strains CC-3403 (RU-387 *nit1 arg7 cw15 mt2*) and CC-125 (137c *nit1 nit2 agg1^+^ mt^+^*) were obtained from the *Chlamydomonas* Resource Center, RRID: SCR_014960 (http://www.chlamycollection.org). Cells were grown in standard Tris-acetate-phosphate (TAP) medium (Gorman and Levine 1965), optionally supplemented with 100 µg/ml L-arginine (TAP-Arg) under continuous cool fluorescent white light of 40–60 µE m^−2^ s^−1^ at 110 rpm at 22°C or alternatively in synchronized cultures with cycles of 14 h at 25°C under light and 10 h at 18°C in darkness.

### Algal genotoxic sensitivity assays

Sensitivity to zeocin (Zc) was examined by scoring cell growth on TAP plates supplemented with 2–4 µg/ml Zc (Sigma-Aldrich) under continuous cool fluorescent white light of 40–60 µE m^−2^ s^−1^. Cells were counted before plating and the same suspension of wt cells and mutants were respectively plated with TAP or TAP supplemented with Zc. Visible colonies appeared after a growth period 8–10 days. The experiments were repeated at least three times.

### DNA preparation and Cas protein purification

The circular plasmid DNA used in the *Chlamydomonas* transformation was isolated from XL-1 blue *Escherichia coli* cells and then column purified according to the manufacturer’s instructions (Machery-Nagel NucleoSpin Plasmid EasyPure). *Streptococcus pyogenes* SpCas9 and *Lachnospiraceae bacterium* LbCas12a (Cpf1) protein was expressed and purified as described in the following six steps (Gagnon *et al.* 2014; Greiner *et al.* 2017): (1) either the SpCas9, LbCas12a expression plasmid pET-28b-Cas9-His (Addgene plasmid, 47327), or p6His-MBP-TEV-huLbCpf1 (Addgene plasmid, 90096), respectively, was transformed into *E. coli* strain Rosetta2 (DE3). (2) The Cas-expressing clone was grown in 500 ml LB medium with 100 µg/ml ampicillin at 37°C for 2 h before induction with 1 µM IPTG. (3) Expression was performed overnight at 25°C. *E. coli* cells were resuspended in lysis buffer (20 mM Tris, pH 8.0, 300 mM NaCl, 10 mM imidazole, DNaseI, and 0.1 mM PMSF) and lysed using an EmulsiFlex-B15 homogenizer (Avestin). (4) The lysate was purified by immobilized affinity chromatography (5 ml nickel column FF-Crude, Desalt 16-60; GE Healthcare). (5) Protein concentration was determined by A260/A280 absorption, diluted to 10 µM (∼3.1 µg/ml) in 1× Buffer O (BO5: Thermo Fisher Scientific), and filter sterilized. (6) Finally, the aliquots were shock frozen in liquid nitrogen and stored at −80°C.

### Cell growth, heat shock, and transformation of *Chlamydomona*s cells

Transformation was performed according the protocols published by Greiner *et al.* (2017). Cells were grown under a synchronized light (14 h, 25°C)/dark (10 h, 18°C) cycle for at least 10 days and kept in exponential growth phase by diluting 1:50 every 3–4 days with fresh TAP(-Arg) medium. At the end of the light phase, cells from a concentration with a density of 1–3 × 10^6^ cells/ml were harvested by centrifugation at 2000 g for 10 min at room temperature (RT, 22°C) and resuspended in MAX Efficiency transformation medium (A24229; Thermo Fisher Scientific) that was supplemented with 40 mM sucrose to a density of 10^8^ cells/ml. Prior to transformation, concentrated cells were heat-shocked at 40°C for 30 min in a thermomixer (Eppendorf) set to 350 rpm. Transformations were performed via electroporation using NEPA21 electroporator (Nepa GeneCo) according to the manufacturer’s instructions and the protocols provided by Yamano *et al.* (2013). The impedance of a 40–45 µl cell suspension ranged between 400 and 550 ohms. CC3403-D5 cells were electroporated using two 8-ms/200-V poring pulses at 50 ms intervals with a decay rate of 40% and were followed by five 50-ms/20-V polarity-exchanged transfer pulses at 50 ms intervals with a decay rate of 40%. CC-125 were electroporated using one 8-ms/300-V poring pulses at 50 ms intervals with a decay rate of 40% and were followed by five 50-ms/20-V polarity-exchanged transfer pulses at 50 ms intervals with a decay rate of 40%.

### Complex of recombinant Cas ribonucleoprotein

Either the recombinant SpCas9 or LbCas12a protein was complexed with guide RNA (gRNA) to form an ribonucleoprotein (RNP). SpCas9 gRNAs were ordered in the following sequence as two RNAs, the scaffold RNA (tracrRNA) with constant sequence, and the target sequence (crRNA). These differed for each target site according to the guidelines of a commercial supplier (Alt-R CRISPRCas9 system; Integrated DNA Technologies). LbCas12a gRNA was used for the cleavage of mut-*aphVIII* with the sequence AAUUUCUACUAAGUGUAGAUGGGAGGCCCCCUCCGCCAUGAGC (spacer underlined) and was ordered from Integrated DNA Technologies. All target (protospacer) sequences used are shown in Supplementary Table S5. Equimolar amounts of tracrRNA and crRNA for SpCas9 or gRNA for LbCas12a were annealed in DUPLEX buffer (100 mM potassium acetate and 30 mM HEPES, pH 7.5; Integrated DNA Technologies) to a final concentration of 10 µM. This concentration was achieved by heating to 95°C for 2 min, which was followed by cooling at a rate of 0.1°C/min. Purified 10 µM Cas9 protein was mixed with equimolar amounts of annealed gRNA in 1x Buffer O (Thermo Fisher Scientific) to a final concentration of 3 µM each and was incubated for 15 min at 37°C. Cells were mixed with 3 µl of 3 µM Cas9RNP. If necessary, both donor DNA for HDR and 0.3 µg (0.1 pmol) of the marker pPmR plasmid (earlier designated pAPHVIII, Greiner *et al.* 2017) were added for preselection of transformants.

### HDR donor strategies

Three types of HDR donors were used: (1) linear ssODNs, (2) linear double-stranded DNA (dsDNA), and (3) plasmids. Oligonucleotides, both 90- and 100-bp ssODNs, were ordered with three phosphorothioate-protected bonds at each end. In the case of a short double-stranded HDR donor, equimolar sense and antisense oligonucleotide were annealed in 1× duplex buffer (100 mM potassium acetate and 30 mM HEPES, pH 7.5 by incubating at 95 °C for 2 min). This was followed by cooling at a rate of 0.1°C/min [Integrated DNA Technologies (https://eu.idtdna.com/pages)]. dsODNs formation was confirmed by a gel image. In total, 40 pmol single-stranded or a mixture of either complementary single-stranded oligonucleotides (20 pmol each), 20 pmol annealed double-stranded oligonucleotides, or 2 µg circular donor plasmid that were used during the transformations. Preliminary experiments demonstrated that varying the amount of ssODN in the range of 20 – 40 pmol did not affect the transformation efficiency. All donor sequences used are listed in Supplementary Table S3.

### Recovery, plating, and picking of the transgenic strains

After electroporation and prior to plating, the cells were diluted in 500 µl of TAP(-Arg) and incubated at 22°C for 24 h. Before plating, cells were counted, and an equal number of cells were plated on a selective medium. In the case of paramomycin selection via mut-*aphVIII* repair, cells were plated with a concentration 10 µg/ml of paromomycin. In the case of hygromycin selection, which was used in transformations with the selective plasmid pAPHVII conferring resistance to hygromycin, selection plates contained 20 µg/ml of hygromycin.

For 2-fluoroadenine (2-FA) selection via the adenine phosphoribosyltransferase (APT) disruption, plates contained 150–200 µg/ml 2-FA. Colonies generally appeared after 7–10 days and were picked with sterile toothpicks before being transferred to 96-well plates containing 180 µl of TAP-Arg.

### Algal mutant screening procedures

PCR amplification of genomic *Chlamydomonas* DNA was performed in a 96-well format using Phire Plant Direct PCR Master Mix (Thermo Fisher Scientific; dilution buffer protocol) and crude cell extracts. Cells were grown in 180 µl of TAP(-Arg) medium in 96-well plates until all wells had turned uniformly green in color. A 40-µl aliquot of each cell culture was then transferred to a 96-well V-bottom culture plate and centrifuged at 2000 g for 10 min at RT. The supernatant was removed, and the pellet was thoroughly resuspended in 20 µl of dilution buffer, incubated for 5 min at RT, centrifuged again at 4000 g for 10 min at RT, and used for PCR. PCR was performed according to the manufacturer’s instructions and according to the protocols of Greiner *et al.* (2017). Oligonucleotide sequences used for screening are listed in Supplementary Table S4.

### *APT* disruption experiments in *Chlamydomonas*

In *APT* disruption experiments, the CC-125 strain and its *ku80* and *polQ* mutants were used as listed in Supplementary Table S1. The gRNA for *APT* (Supplementary Table S5) was designed using the CRISPOR website (http://crispor.tefor.net/; Haeussler *et al.* 2016). In total, 9 pmol *APT* Cas9/gRNARNP and 40 μl heat-shocked cells were used per transformation. Cells were recovered at RT (22°C) for 24 h. Cells were selected for plating on plates containing 150–200 µg/ml 2-FA. Colonies appeared after 8–10 days.

### The mut-*aphVIII* repair assay transformations in *Chlamydomonas*

In mut-*aphVIII* repair assay transformations, the CC3403-D5 strain (earlier designated CC-3403-GTS3) and its *ku80* and *polQ* mutants were used as listed in Table S1. For transformations we used 9 pmol *EMX1* Cas9/gRNA RNP or 9 pmol mut-*aphVIII* LbCas12a/gRNA RNP and different donor DNA including: (1) 2 µg (0.8 pmol) of pHDR-APHVIII^Δ120^ (circular); (2) 40 pmol single-stranded oligonucleotide template that matched the protospacer strand (P-ssODN); (3) 40 pmol single-stranded oligonucleotide template that matched the spacer strand (S-ssODN); (4) a mixture 20 pmol P-ssODN plus 20 pmol P S-ssODN without annealing; (5) 20 pmol annealed P-ssODN/S-ssODN. We used 40 μl (4 × 10^6^) of heat-shocked (40°C, 30 min) cells per transformation. Donor ssODNs were either 100 nt or 100 bp in length, with 5′ and 3′ regions, 45 and 55 nucleotide (nt)/bp long, respectively, which were homologous to *APHVIII* sequences flanking the transgenic insert in the mut-*aphVIII* construct (Supplementary Figure S3). Similar, pHDR-APHVIII^Δ120^ included the 1350 bp fragment with the 5′ and 3′*APHVIII* specific homology arms, 503 and 847 bp long, respectively (Greiner *et al.* 2017; Supplementary Table S3 and Figure S3). After transformation, cells were recovered with RT (22°C) for 24 h. Cells were counted, and the same number of cells were plated on selective medium supplemented with paromomycin (10 µg/ml). Colonies appeared after 8–10 days. During experimentation, we found that a change in the amount of ssODN in the range of 20–40 pmol did not affect the number of PmR clones produced.

### The *SNRK2.2* disruption experiments in *Chlamydomonas*

In *SNRK2.2* disruption experiments, the CC-125 strain and its *ku80, ku70*, and *polQ* mutants were used as listed in Table S1. The gRNA for *SNRK2.2* was designed using the CRISPOR website (http://crispor.tefor.net/; Haeussler *et al.* 2016; Supplementary Table S5). For transformations we used 9 pmol *SNRK2.2* Cas9/gRNA RNP and a mixture containing 0.3 µg (0.1 pmol) of the marker pPmR plasmid (circular) and different donor DNA including: (1) 2 µg (0.7 pmol) pSNRK-FLAG (circular); (2) 40 pmol single-stranded oligonucleotide template containing the integrated FLAG sequence that matched the protospacer strand (P-ssODN-FLAG); (3) 40 pmol single-stranded oligonucleotide template containing the integrated FLAG sequence that matched the spacer strand (S-ssODN-FLAG); (4) a mixture 20 pmol P-ssODN-FLAG plus 20 pmol P S-ssODN-FLAG without annealing. We used 40 μl (4 × 10^6^) of heat-shocked (40°C, 30 min) cells per transformation. ssODNs contained the *SNRK2.2* specific 33 and 28 nt homology arms, as well as the plasmid pSNRK-FLAG, which comprised 1113 and 770 bp homology arms flanking the FLAG sequence (Supplementary Table S3). The 29 nt FLAG sequence contained an in-frame stop codon and altered the reading frame. Cells were recovered at RT (22°C) for 24 h. Cells were counted before plating, and the same number of cells were plated on TAP medium supplemented with paromomycin (10 µg/ml). Colonies appeared after 8–10 days and were used for green-blue screening of mutants defective in the *SNRK2.2* gene.

### Blue-green screening assay in *Chlamydomonas*

Mutants defective in the *SNRK2.2* gene were phenotypically screened by an arylsulfatase assay (Davies *et al.* 1992). *SNRK2.2* (SNF1-related protein kinase) has been observed to repress -S-inducible genes, including arylsulfatases *ARS1* and *ARS2*. In TAP medium (no sulfur limitation), wt *Chlamydomonas* cells have been found to not express arylsulfatase. When *SNRK2.2* is absent, it is expected that arylsulfatases will also be expressed in +S conditions. The presence of arylsulfatase can be visualized with 5-bromo-4-chloro-3-indolyl sulfate (X-SO4), as this dye forms a blue precipitate when the sulfate group is cleaved off. Since arylsulfatase is secreted, it is not necessary to disrupt cells to obtain measurements. The blue-green screening was done directly on agar plates with living cells by spraying grown colonies with 3 mM X-SO4 until the plate is covered with liquid (∼1 ml). Blue color appeared after 24 h incubation at RT. ‘Blue’ colonies were collected and used in PCR scre ening procedures (Supplementary Figure S7). As a result, there were no blue colonies found in the control experiments where we used SpCas/gRNA-RNPs specific for cleavage of *VGCC, COP1*, or *CHR1* genes. The mutation rate was calculated based on the percent of paromomycin-resistant blue colonies of all PmR colonies, in which the *SNRK2.2* gene was disrupted and confirmed via sequencing.

### Statistical analysis

Data were presented as mean ± SEM and were analyzed using the KyPlot software package and Excel to assess the statistical significance of their differences. Differences were considered significant at *P* < 0.05.

### Data availability

Strains, plasmids, and plasmid sequences are available upon request and will be deposited at the *Chlamydomonas* Resource Center. The authors affirm that all data necessary for confirming the conclusions of the article are present within the article’s text, figures, and tables. All DNA oligonucleotides, sequences of donor DNA, insertions inside mutants, and protospacer sequences used in this study are in Supplementary Tables. Supplementary material is available at figshare: https://doi.org/10.25387/g3.13857209.

## Results

### Generation of *Chlamydomonas* POLQ, KU80, and KU70 deficient mutants

The *C. reinhardtii* genome contains the putative homologs for *POLQ* (Cre16.g664300)*, KU80* (Cre10.g423800), and *KU70* (Cre13.g607500) genes (https://phytozome.jgi.doe.gov/pz/portal.html). To investigate *Chlamydomonas* POLQ, KU80, and KU70 function *in vivo* and understand their role in the repair of CRISPR Cas9-induced double-stranded breaks, we generated *polQ, ku80*, and *ku70* mutants using the CRISPR/SpCas9-mediated targeted gene disruption, as previously described by Greiner *et al.* (2017)*. Chlamydomonas polQ, ku80*, and *ku70* mutants were generated in the wt strain CC-125 or the model cell-wall deficient strain CC3403-D5, which contains a defective paromomycin resistant (mut*-aphVIII*) gene (Greiner *et al.* 2017). Transformation mixtures were used containing the following three components: (1) a gene-specific SpCas9/gRNA RNP complex (Supplementary Table S5), (2) a mixture of donor complementary gene-specific (POLQ or KU80 or KU70) ssODN repair templates encoding a FLAG epitope (Supplementary Table S3), and (3) the selective plasmid pAPHVII conferring resistance to hygromycin (Greiner *et al.* 2017). All mutants recovered contained long insertions, which included premature stop codons inside the first exons and a shifted reading frame that should result in complete elimination of gene functions in all mutants. These insertions typically ranged from 30 to 371 bp, but were more than 3 kb in the case of the mutant ΔPOLQ-E2. They included FLAG sequences from the DNA repair template and different fragments of the selective plasmid (Supplementary Table S1). All the alleles described in Supplementary Table S1 are likely to constitute null alleles. The described mutations have been stable for > 2 years since their generation. Growth curves of the parental CC-125 strain and mutants in TAP liquid media (Supplementary Figure S1) show that KU80, KU70, and POLQ deficiencies do not significantly affect the growth rate of these mutants.

We first confirmed that inactivation of *KU80*, *KU70*, and *POLQ* caused the expected sensitivity to the drug Zc which creates DSBs (Ehrenfeld *et al.* 1987; Chankova *et al.* 2007; Chen *et al.* 2008; Čížková *et al.* 2019). Surprisingly, *polQ* cells were found to be more sensitive to Zc than *ku80* and *ku70* cells. When 10^5^ cells were plated on medium with 4 μg/ml Zc, only 4–8 *polQ* colonies and about 2 × 10^3^ colonies of *ku80* or *ku70* mutants were found to grow (Supplementary Figure S2A). A spot test with diluted cell suspensions also indicated a higher sensitivity for the *polQ* mutant to Zc in comparison to *ku80* and *ku70* cells (Supplementary Figure S2B). These results suggested that TMEJ may be the main pathway for DSB recovery. Plecenikova *et al*. (2014) found that the wall-less strain 302, which carried a long deletion in chromosome 16 (including the *POLQ* gene), exhibited a strong sensitivity to Zc. This finding is consistent with our data suggesting the high sensitivity of the *polQ* mutants of the parental strain CC-125 to Zc.

### DNA template-free repair of CRISPR-Cas9-induced genomic DSB

**Figure 1:**
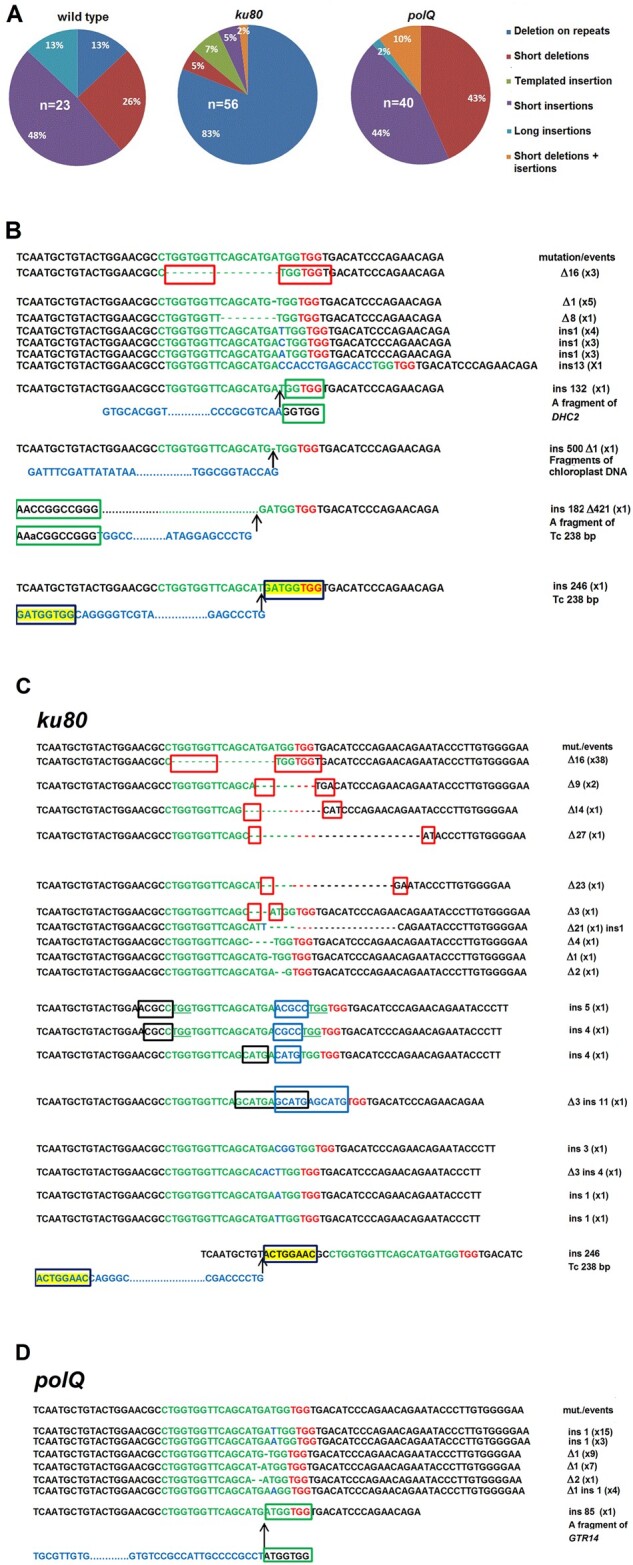
Types of mutations generated in the *APT* gene by repair of SpCas9-mediated DSB in wt, *ku80*, and *polQ* strains. (A) Ratio of the different types of mutations found in wt (*n* = 23); ΔKU80-C4 (*n* = 43) and ΔKU80-C6 (*n* = 13) (ku80); ΔPOLQ-E2 (*n* = 8) and ΔPOLQ-G2 (*n* = 32) (polQ) strains. *n*, number of analyzed mutants in the indicated strains. (B) Sequences of mutations found in wt strain. (C) Sequences of mutations found in *ku80* strains. (D) Sequences of mutations found in *polQ* strains. Green letters indicate the sequence recognized by the sgRNA (protospacer). The PAM is shown in red. DNA insertions are shown in blue, whereas deletions are shown with dashes. In the red frames, the microhomologies potentially involved in TMEJ-mediated DSB repair are depicted by dashes as result in deletions. Dashes of difference colors indicate what kind of a sequence (protospacer, PAM, or flanking) was deleted. In the blue frames, templated insertions are shown by black frames. In the green frames, microhomologies believed to be involved in insertions of chromosome DNA fragments are shown. Microhomology arising from the introduction of a 238 bp transposon is shown in green frames on a yellow background. The length of deletions (shown as Δ) or insertions (shown as ins), as well as the source of inserted DNA, is indicated to the right. Numbers in parenthesis indicate the number of observed colonies with the particular mutation.

To investigate the impact of the error-prone cNHEJ and TMEJ pathway in repair of CRISPR/Cas9-induced DSB, we chose to target the endogenous and selectable *APT* gene. The *APT* gene encodes the protein participating in so-called purine ‘salvage pathways’. These pathways recycle purine compounds for nucleotide biosynthesis (Ashihara *et al.* 2018). Guzmán-Zapata *et al.* (2019) found that the toxic adenine analog, 2-FA, entered *Chlamydomonas* cells and was metabolized by APT, thus leading to cell death. Inactivation of the *APT* gene by SpCas9-mediated gene disruption was found to result in the increased resistance of *Chlamydomonas* mutants to 2-FA (Guzmán-Zapata *et al.* 2019). We targeted the *APT* exon 1 locus using Cas9 RNPs and sequenced 2-FA-resistant clones obtained in wt cells, *ku80*, and *polQ* mutants. We hypothesized that repair events in POLQ-deficient cells would reflect cNHEJ events, whereas repair events in KU80-deficient cells be through TMEJ. The knockout efficiency in 2-FA-resistant clones of wt, *ku80*, and *polQ* strains detected by sequencing *APT* exon 1 ranged from 11% to 83% in independent experiments. This wide variation in the percentages is likely due to transposon insertions within exon 3 or other positions that we did not analyze (unpublished data; Guzmán-Zapata *et al.*, 2019). Almost all of the 121 mutations generated using Cas9 RNPs in the wt cells and mutants occurred at position 3 or 4 bp before the protospacer adjacent motif (PAM), which is consistent with what other researchers have expected for SpCas9 cleavage (Zuo and Liu 2016). Types and sequences of mutations are depicted in Figure 1 and Supplementary Table S2). Two of 121 analyzed mutants have revealed that they contain insertions of the 238 bp complete copies of a Gulliver-related transposon (CR165; GenBank: DQ446208.1) into position 5 or 31 bp upstream of PAM (Figure 1, shown noncoding DNA strand) accompanying by duplication of the neighboring 8-bp target sequence as seen by Kim *et al.* (2006) in other strains.

Analysis of 23 mutations generated in the CC-125 wt strains discovered that more than a half of modifications (15/23) were the 1 bp deletions/insertions that resembled products of DSB repair through cNHEJ pathway (Figure 1, A and B). A few base pairs deletions/insertions at a repaired DSB are known as typical modifications produced by the canonical cNHEJ (Bétermier *et al.* 2014). Other identified mutations (3/23) were the 16 bp deletions occurring between the microhomology (TGGTGGT) flanking the predicted DSB which could be produced by TMEJ pathway. Microhomology-mediated deletions were found to be specific characteristics of TMEJ products (Black *et al.* 2016; Schimmel *et al.* 2017). The remaining five mutations included a 8 bp deletion, a 10 bp insertion and three unexpectedly long insertions (from 132 to 500 bp) which were comprised of the following potential three fragments of nuclear or chloroplast DNA: (1) the 132 bp Dynein heavy chain 2 fragment (*DHC2*, Cre09.g392282) gene, (2) jointed chloroplast DNA fragments (500 bp), or (3) the 182 bp fragment of the transposon Tc 238 bp, which was combined with a 421 bp deletion (Supplementary Table S2). The most likely source of DNA for these long inserts could be the *Chlamydomonas* DNA that was extracted from cells in the culture medium, which lysed before or during electroporation (Zhang *et al.* 2014). Interestingly, the *DHC2* and Tc 238 bp fragments to be inserted were flanked by microhomology with APT sequences adjacent to DSB (shown in green frames in Figure 1B).

A similar analysis was performed for 56 mutations found at the repaired SpCas9 cleavage site in KU80 deficient mutants. As shown in Figure 1A, we discovered that 86% mutations were deletions in the 1–23 bp range, of which almost all (96%) took place between the 2–7 bp repeated sequences immediately upstream and downstream of the DSB. Most of these deletions (38/44) occurred between the longest repeats of 7 bp. Another distinct feature of *ku80* mutants was the presence of so-called templated insertions (shown by black frames, Figure 1B) at the repair junction where flanking DNA served as a template for repair reactions (shown by blue frames, Figure 1B). As microhomology-mediated deletions and templated insertions are characteristic features of DSB repair through TMEJ (Black *et al.* 2016; Schimmel *et al.* 2017), their presence in 90% analyzed mutants is evidence for the involvement of POLQ in DSB repair in KU80 deficient *Chlamydomonas* cells. In *ku80* mutants, we also found a few (4/56) short insertions/deletions and the insertion of a copy of Tc 238 transposon (Figure 1C). Notably, the spectrum of SpCas9-induced mutations identified in POLQ deficient mutants clearly differed from those found in wt and *ku80* cells (Figure 1). Thirty nine of forty analyzed *polQ* mutants revealed the 1 bp deletions, insertions, or exchanges (resulting from combination of deletion and insertion of 1 bp). As mentioned earlier, these types of DNA modification are specific for DSB repair through cNHEJ pathway. Only 1 of 40 analyzed *polQ* mutants contained an integrated 85 bp fragment of the glycosyltransferase *GTR14* gene (Cre14.g616200.t1), which possessed a 7 bp microhomology to the *APT* region adjacent to DSB (shown in Figure 1B in the green frame). Similar microhomologies were also seen for the two long insertions discussed earlier. A comparison between wt cell, *ku80* mutants, and *polQ* mutants has revealed that wt cells contain typical mutation signatures of both cNHEJ and TMEJ, with the predominant number of short insertion/deletions (Figure 1A). In wt cells, cNHEJ and TMEJ act in parallel, which creates mutation spectra that include 1-nt deletions/insertions specific for cNHEJ and deletions between microhomology regions specific for TMEJ.

### SpCas9-mediated precise HDR in a model selectable transgene

To assess the effect of KU80, KU70, and POLQ deficiency on DSB repair outcome we tested the repair of an integrated defective paromomycin resistance marker (mut-*aphVIII*), which can be monitored through the formation of PmR resistant clones (Supplementary Table S1 and Figure S3). We compared repair outcomes resulting from using (1) single-stranded oligonucleotide templates that matched either the protospacer strand (P) or the spacer strand (S) of the *APHVIII* gene; (2) a double-stranded oligonucleotide template; or (3) a pHDR-APHVIII^Δ120^ plasmid containing the 503 + 847 bp homology arms (Supplementary Table S3). Successful, template-mediated repair results in the restoration of a functional paromomycin resistance gene. ssODN templates (both S-ssODN and P-ssODN) produced the lowest repair efficiencies. A mixture of unannealed S-ssODN+P-ssODN, annealed S-ssODN/P-ssODN, and pHDR-APHVIII^Δ120^ plasmid all produced higher repair efficiencies (Supplementary Figure S4). Notably, the amount of plasmid DNA (0.8 pmol) was significantly lower than that in ssODN experiments (40 pmol; Supplementary Figure S4). A similar or slightly larger number of target mutants was found without complementary ssODN annealing in comparison to the annealed dsODN (the ± 95% CI were 188 ± 74, 100 ± 44), which demonstrates that annealing can be omitted. Our results suggest that ssODNs operate independently in these processes. These inferences need to be verified with additional combinations of gRNAs, loci, and repair templates to better assess their generality.

**Figure 2:**
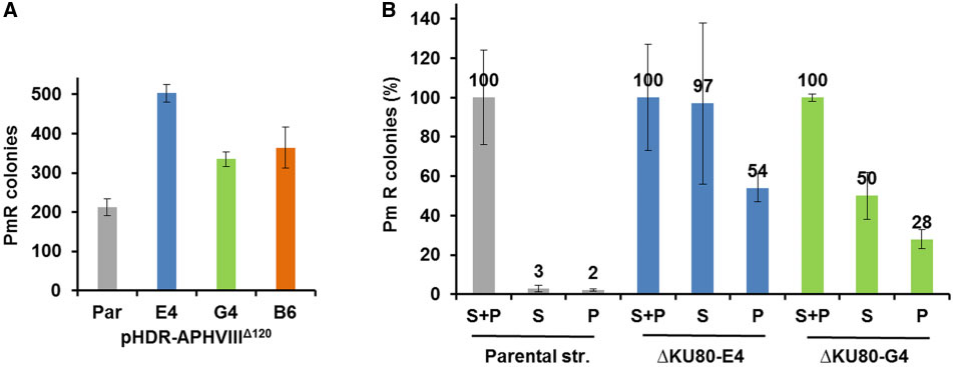
Effect of KU80 or KU70 deficiency and repair template on HDR of a mut-aph*VIII* transgene. (A) Repair efficiency using single-stranded protospacer strand (P), spacer strand (S) repair templates, or an unannelaed mixture of the two (S + P) in cells of the indicated genotype. Efficiency of repair is presented as the percentage of the number of PmR clones obtained with the indicated repair template, normalized with the data obtained from their mixture S + P (the original data are in Supplementary Figure S5). (B) Efficiency of repair using a pHDR-APHVIII^Δ120^ plasmid using *EMX1* mut-*aphVIII* SpCas9 RNP in the parental CC3403-D5 (Par) and mutants D5-ΔKU80-E4 (E4), D5-ΔKU80-G4 (G4), ΔKU70-B6 (B6). Efficiency of repair is presented as the number of PmR clones obtained by electroporation of cells with RNP and DNA. In all experiments, the number of cells was counted before plating, and the same number of cells were plated on TAP medium supplemented with Pm, 10 μg/ml. Error bars represent SEM (*n* = 6 separate experiments for the parental CC3403-D5 strain in (B) and *n* = 3 for the other categories). HDR events from individual single-stranded repair templates in the parental strain were found to be statistically different from HDR events in the KU80 deficient mutants (*P* < 0.05).

**Figure 3:**
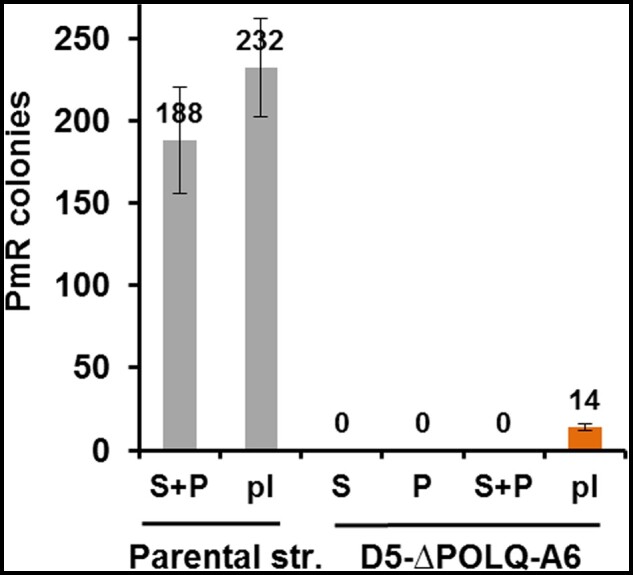
Effect of POLQ deficiency and repair template on HDR of an integrated mut-*aphVIII* transgene. Efficiency of repair is presented as the number of PmR clones obtained by electroporation of cells with *EMX1* RNP and either a single-stranded protospacer strand (P), spacer strand (S) repair templates, an unannealed mixture of the two (S + P) or the pHDR-APHVIII^Δ120^ plasmid (pl) in cells of the indicated genotype. In all experiments, the number of cells were counted before plating, and the same number of cells were plated on TAP medium supplemented with Pm, 10 μg/ml. Error bars represent SEM (*n* = 6 separate experiments for each category). HDR events from pHDR-APHVIII^Δ120^ in the parental strain were found to be statistically different from HDR events in the POLQ deficient mutants (*P* < 0.01).

Next, we assessed the impact of NHEJ and POLQ on repair. When using ssODN templates for KU80 deficient mutant, we found a clear stimulation of the mut-*aphVIII* repair compared with the parental strain (Figure 2A and Supplementary Figure S5). In the parental strain the number of PmR target mutants obtained with S-ssODN or P-ssODN ssODN template was in more than 30-fold lower than with a mixture S-ssODN+P-ssODN. However, the KU80 deficiency resulted in a sharp increase in the number of PmR target mutants obtained with any of ssODNs. As shown in Figure 2A, in the *ku80* mutants the efficiency of the mut-*aphVIII* repair for S-ssODN or P-ssODN was in the range of 28–97% of that found for a S-ssODN + P-ssODN mixture. In transformations with the best repair templates for parental cells (unannealed mixture of S-ssODN+P-ssODN and the pHDR-APHVIII^Δ120^ plasmid), we did not detect a stimulation of mut-*aphVIII* repair in KU80 and KU70 deficient mutants (Supplementary Figure S4). For the S-ssODN+P-ssODN mixture, HDR events in ku80 mutants were statistically different (*P* < 0.05) compared with the parental strain only in D5-ΔKU80-G4 cells, but not in D5-ΔKU80-E4 strain (Supplementary Figure S5). For pHDR-APHVIII^Δ120^ plasmid, a statistically significant stimulation (*P* < 0.05) of the mut-*aphVIII* repair was found in D5-ΔKU80-E4 and D5-ΔKU80-G4 cells, but not in D5-ΔKU70-B6 cells (*P* < 0.1; Figure 2B). Interestingly, POLQ deficiency completely eliminated the homology-directed mut-*aphVIII* repair with the use of ssODN templates. However, they still enabled mut-*aphVIII* repair with the pHDR-APHVIII^Δ120^ template (Figure 3). For all conditions, DNA sequencing of randomly chosen PmR clones confirmed the precise repair of the mut-*aphVIII* gene. The suppression of mut-*aphVIII* repair using ssODN templates was confirmed in experiments with LbCas12a (Supplementary Figures S3 and S6). The high functional activity of LbCas12a (Zetsche *et al.* 2015) RNP in the *Chlamydomonas* wall-less cw15 strain was demonstrated by Ferenczi *et al.* (2017). The number of PmR clones generated using the pHDR-APHVIII^Δ120^ template was considerably lower (the ± 95% CI was 14 ± 3.6) for the POLQ deficient strain D5-ΔPOLQ-A6 than those that were found for parental wt strain or its KU80 deficient mutants (>200) containing the native *POLQ* gene (Figures 2 and 3). DNA sequencing of randomly chosen PmR clones confirmed the precise repair of the mut-*aphVIII* gene. We suggest that POLQ activity is important for repair with both the ssODN repair templates and templates containing long gene-specific homology arms. Presented findings also show that KU80 deficiency results in stimulation of the SpCas9-mediated precise mut-*aphVIII* repair, as detected by direct selection of homology-directed recombinants.

### SpCas9-mediated homology-directed inactivation of a non-selectable gene

To overcome the low rate of SpCas9-mediated HDR in non-selectable genes, we targeted the nonessential *SNRK2.2* gene (Cre12.g499500), which encodes for SNF1-related protein kinase. We also used donor repair templates that were supplemented with plasmids containing antibiotic resistance markers (Greiner *et al.* 2017). By selecting transformants that were resistant to antibiotics, we were able to isolate clones that were enriched with the desired mutations in non-selectable genes. Jinkerson and Jonikas (2015) had demonstrated that drug resistance cassettes randomly integrated into the *Chlamydomonas* genomic double stranded breaks that occur during transformation. These insertions were without obvious hot or cold spots, which have been presumed to have happened through the cNHEJ pathway. This even distribution has been used to prepare large collections of insertion mutants (Salome and Merchant 2019). We first tested the effect of DNA-repair deficiency on random and targeted insertions for a selective plasmid, as well as repair templates, in the wt strain CC-125 and its *ku80, ku70, and polQ* mutant derivatives.

**Figure 4:**
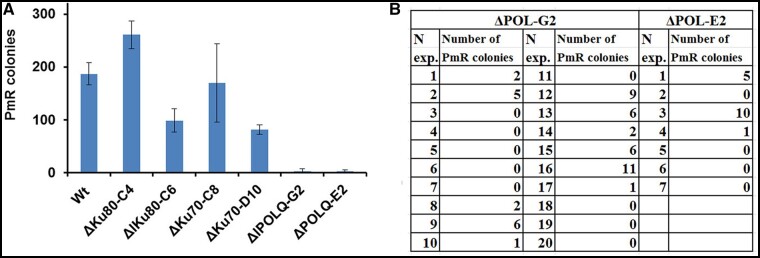
Random insertions of the pPmR plasmid in KU80, KU70, and POLQ deficient mutants. Shown is the efficiency of random insertions of the circular pPmR plasmid, which was used for preselection of transformants, in the parental wt strain and its KU80, KU70, and POLQ deficient mutants. Transformation efficiency is presented as the number of PmR colonies obtained by electroporation of cells with pPmR plasmid (0.3 µg = 0.1 pmol) plus *SNRK2.2* RNP and single-stranded repair templates (ssODN-FLAG). (A) Random insertions of pPmR in the parental CC-125 (wt) and mutants ΔKU80-C4, ΔKU80-C6, ΔKU70-D10, ΔKU70-C8, ΔPOLQ-E2, and ΔPOLQ-E2. Error bars represent SEM for separate experiments in each category. The number of experiments in each category is listed as: wt, *n* = 13; ΔKU80-C4, *n* = 20; ΔKU80-C6, *n* = 6; ΔKU70-D10, *n* = 4; ΔKU70-C8, *n* = 3; ΔPOLQ-G2, *n* = 20; ΔPOLQ-E2, *n* = 7. (B) Random insertions of pPmR in mutants ΔPOLQ-E2 and ΔPOLQ-E2. The table shows the number of PmR colonies obtained in separate experiments. In all experiments, the number of cells was counted before plating, and the same number of cells was plated on TAP medium supplemented with Pm, 10 μg/ml.

Random insertion was evaluated by the number of paromomycin resistant clones generated with the co-transformed pPmR plasmid, which contains the *APHVIII* gene (Figure 4A). Upon transformations of ΔKU80-C4, ΔKU80-C6, ΔKU70-C8, ΔKU70-D10 cells using 0.1 pmol of pPmR the random integration was not statistically different from wt cells (Figure 4A). We did not find insertions of pPmR into the SpCas9 cleavage site in more than 100 randomly selected PmR colonies of either the wt or *ku80* cells. In contrast, in ΔPOLQ-G2 and ΔPOLQ-E2 cells, we found only a residual level of random integration (Figure 4).

**Figure 5:**
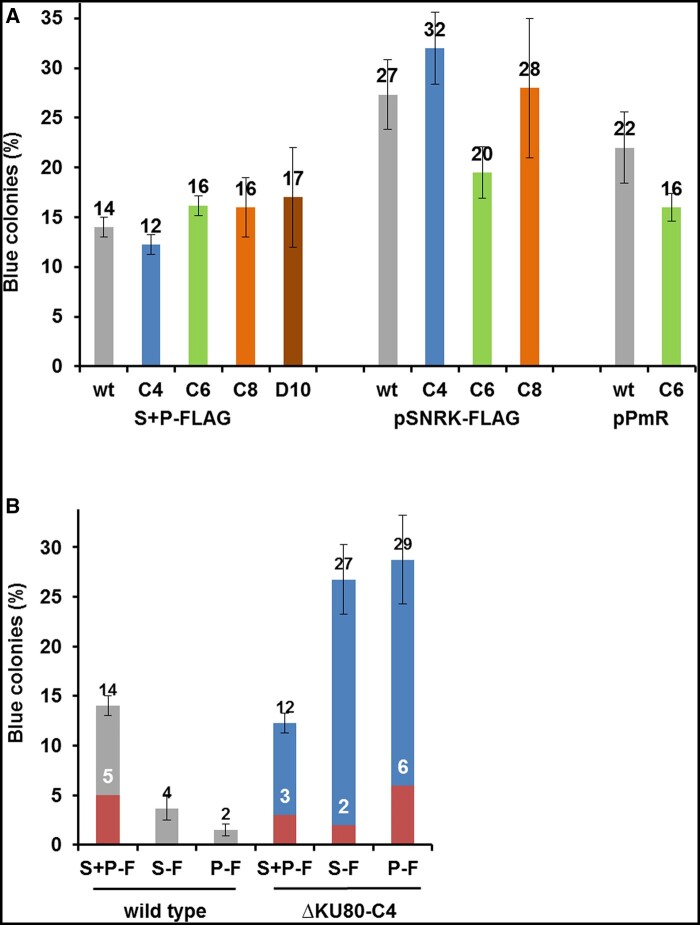
SpCas9-induced targeted inactivation of the non-selectable *SNRK2.2* gene. The efficiency of *SNRK2.2* inactivation upon CRISPR/SpCas9-induced double-strand breaks with the indicated templates is shown. Mutants that were defective in the *SNRK2.2* gene were phenotypically screened for blue coloration by an arylsulfatase assay (Davies *et al.* 1992). Genotypes tested were wt CC-125 cells (wt) and mutants ΔKU80-C4 (C4), ΔKU80-C6 (C6), ΔKU70-C8 (C8), and ΔKU70-D10 (D10). PmR colonies were obtained by cell electroporation with *SNRK2.2* RNP, pPmR (0.3 µg = 0.1 pmol), and donor DNA templates. The following donor DNA templates were used: (A) a mixture of spacer ssODN-FLAG + prospacer ssODN-FLAG (S + P-FLAG), pSNRK-FLAG (2 µg = 0.7 pmol) or only pPmR (4 µg = 1.4 pmol); (B) protospacer ssODN-FLAG (P-F), spacer ssODN-FLAG (S-F), a mixture spacer ssODN-FLAG + prospacer ssODN-FLAG (S + P-F). Efficiency is presented as the percentage of blue colonies found in all PmR colonies. Percentage of colonies containing the correctly integrated FLAG is highlighted in red. Error bars represent SEM for separate experiments in each category. The number of experiments in each category is listen as: (A) For S + P-FLAG: wt, *n* = 3; ΔKU80-C4, *n* = 20; ΔKU80-C6, *n* = 12; ΔKU70-C8, *n* = 5, ΔKU70-D10, *n* = 4. For pSNRK-FLAG: wt, *n* = 3; ΔKU80-C4, *n* = 8; ΔKU80-C6, *n* = 2; ΔKU70-C8, *n* = 2. For pPmR wt, *n* = 5; ΔKU80-C6, *n* = 2. (B) For wt: S + P-F, *n* = 3; S-F, *n* = 3; P-F, *n* = 3. For ΔKU80-C4: S + P-F, *n* = 12; S-F, *n* = 4; P-F, *n* = 4. For individual single-stranded repair templates the targeted *SNRK2.2* inactivation events in wt cells were statistically different from that in ΔKU80-C4 cells (*P* < 0.01). In all experiments, the number of cells was counted before plating, and the same number of cells was plated on TAP medium supplemented with Pm, 10 μg/ml.

We next wished to determine the role of NHEJ and POLQ in homology-directed inactivation of non-selectable genes. For this purpose, the “blue-green” assay of arylsulfatase directly on agar plates was adapted (Davies *et al.* 1992). Homology-directed targeting in a non-selectable gene was tested through targeted inactivation of the sulfur acclimation gene *SNRK2.2* (Cre12.g499500) due to site-specific integration of the 29 bp fragment (FLAG) containing in-frame stop codon. SNRK2.2 deficiency of preselected PmR cells resulted in expression of arylsulfatase in sulfur-containing TAP medium. Arylsulfatase activity can be visualized with a chromogenic substrate forming a blue precipitate when the sulfate group is cleaved off (Supplementary Figure S7). We isolated blue colored colonies contained *snrk2.2* cells, which were then used for comparison of precise and imprecise repair outcomes between wt cells and repair mutants. In transformations of either KU80 or KU70 deficient cells with oligos S-ssODN-FLAG + P-ssODN-FLAG or pSNRK-FLAG repair templates, the percentages of blue PmR clones was comparable with wt cells (Figure 5A). In the case of transformations of wt cells and ΔKU80-C6 cells, we found that an increase in the amount of pPmR from 0.3 to 4 μg (1.4 pmol) resulted in the percentages of blue PmR clones that were in a range similar to that found using gene-specific repair templates (Figure 5A). These data suggests that exogenous DNA without long specific gene homology at the high concentration could integrate into the SpCas9 cleavage site, which is consistent with data from other studies (Shin *et al.* 2016; Kim *et al.* 2020; Picariello *et al.* 2020). In transformations of the wt cells with individual single-stranded DNA templates, S-ssODN-FLAG, or P-ssODN-FLAG plus pPmR (0.1 pmol), we found only 2–4% blue PmR colonies, thereby indicating ineffective targeted integrations of ssODN and/or pPmR when a small amount of plasmid was used. However, in ΔKU80-C4 cells the gene targeting with ssOligos was stimulated, as visualized by 27% for S-ssOligo-FLAG and 29% for P-ssOligo-O blue colonies with destructed *SNRK2.2* gene (Figure 5B).

To test the nature of the recovered mutations, PCR products for *snrk2.2* Exon 1 of blue PmR clones were sequenced and analyzed. At the SpCas9 cleavage site, most of the 40 randomly picked and sequenced blue clones included multiple copies of the FLAG, fragments of ssODN-FLAG, pPmR plasmid, and chromosome regions adjacent to the cleavage site. A complex structure of insertions is exemplified in Supplementary Figure S7C and consistent with data from previous research on other SpCas9-mediated mutations (Greiner *et al.* 2017) and modifications known for inactivating the *KU80*, *KU70*, and *POLQ* genes detected in our mutants (Supplementary Table S1). Although we had a small sample size, the sequencing of generated mutants revealed the similar low percentage for correct FLAG insertions. We observed precise FLAG insertions at a range of 2–6% of the PmR clones generated with individual ssOligos in ΔKU80-C4 cells and with the best repair template S-ssODN-FLAG + P-ssODN-FLAG in ΔKU80-C4 or wt cells (Figure 5B). For *polQ* mutants we did find blue colonies among 30 PmR colonies that we were able to generate by electroporation with a mixture of *SNRK2.2* RNP, pPmR (0.1 pmol) and ssODN templates in more than 20 experiments (unpublished data). Presented data show that KU80 deficiency results in stimulation of SpCas9-mediated homology-directed inactivation of a non-selectable *SNRK2.2* gene through erroneous site-specific FLAG integration when donor ssODN templates were used.

## Discussion

### Efficient generation of *polQ, ku80*, and *ku70* mutants with SpCas9-mediated gene targeting in *Chlamydomona*s

In this study, we analyzed the contribution of the TMEJ and cNHEJ DNA-repair pathways to SpCas9-mediated template-free targeted mutagenesis, HDR and random integration of transgenes in *Chlamydomonas*. Using CRISPR/SpCas9 gene targeting, we created the *ku80, ku70, and polQ* null alleles in wt CC-125 and the transgenic strain CC-3403-D5 containing an inactivated paromomycin resistance gene (mut-*aphVIII*). At first, we phenotypically confirmed that *ku80*, *ku70*, and *polQ* cell lines were defective in DNA repair pathways by assessing their sensitivity to DSB-inducing agent Zc. We found that *ku80* and *ku70* cells were more sensitive to Zc than wt cells but more resistant than *polQ* mutants (Supplementary Figure S2). The high Zc sensitivity of *polQ* mutants of CC-125 strain is consistent with the previously observed high Zc sensitivity of *ΔPOLQ* mutants of wall-less *Chlamydomonas* strain cw15-302 (Plecenikova *et al.* 2014). These observations suggest that in *Chlamydomonas* POLQ is a significant contributor to the repair of SpCas9-induced DSBs. High sensitivity to other DSB-inducing agents (*e.g.*, ionizing radiation, bleomycin, etoposide, neocarcinostatin, doxorubicin, and others) have been demonstrated for *POLQ* deletion in both mouse and human cell lines (Goff *et al.* 2009; Higgins *et al.* 2010; Yousefzadeh *et al.* 2014; Ferreira da Silva *et al.* 2019). Similar phenotypes have been observed in the deletion mutants of NHEJ factor Ligase 4 (ΔLIG4) in animal cells and the moss *Physcomitrella patens* (Adachi *et al.* 2001; Ferreira da Silva *et al.* 2019; Mara *et al.* 2019). Interestingly, human HAP1 cells with defected *ΔLIG4* have been found to be even more sensitive to DSB-inducing agents than *ΔPOLQ* cells, and cNHEJ was considered to be the main pathway for DSB repair (Mara *et al.* 2019).

### In template-free repair of CRISPR/Cas9-induced genomic DSB cNHEJ and TMEJ operate in parallel

In this study, we analyzed the types of mutations caused by the repair of the SpCas9-induced DSB in order to understand which repair pathways underlay this process. Around 90% of the mutations generated in *ku80* mutants with POLQ still present are microhomology-mediated deletions and templated insertions, which have been reported characteristic features of DSB repair through the TMEJ pathway (Black *et al.* 2016; Schimmel *et al.* 2017). In POLQ deficient cells, predominant mutation types have been observed to have 1 nt insertion, deletions and exchanges. It is possible that these could be the result of 1-bp staggered ends produced by SpCas9 and repaired by cNHEJ, which is considered to be not inherently error-prone but adaptable to the structure of the DNA ends (Bétermier et al. 2014; Pawelczak *et al.* 2018; Zuo and Liu 2016). In *Chlamydomonas* wt cells, the mutation profile of repaired SpCas9 cuts included both 1 nt deletions/insertions specific for cNHEJ and deletions between microhomology regions specific for TMEJ. These data suggest that cNHEJ and TMEJ act in parallel. Other organisms appear to have differences in the utilization of TMEJ and NHEJ to repair DSBs. For example, in *C. elegans*, SpCas9-induced mutations are entirely mediated by TMEJ and not by NHEJ (van Schendel *et al.* 2015). Inactivation of genes encoding for cNHEJ key proteins, KU80 or DNA ligase 4 were observed to not change the frequency and the type of SpCas9-mediated mutations in comparison to the wt. POLQ disruption has been found to result in a sixfold decrease of mutagenesis efficiency and very long deletions at target sites (van Schendel *et al.* 2015). POLQ also has been seen to play a major role in the repair of CRISPR/Cas-induced DSB in the moss *Physcomitrella patens* (Mara *et al.* 2019) and *Arabidopsis* (Shen *et al.* 2017). In *P. patens* wt cells and cNHEJ-deficient *lig4* mutants have been found to have a similar high mutagenic rate and mutation spectra, including microhomologies and template insertions characteristic for TMEJ. Human HAP1 wt and polQ mutant cell lines have been observed to have very similar insertions/deletions profiles, which were distinct from the larger deletions seen in *ku80* cells (Ferreira da Silva *et al.* 2019). This finding indicates that in mammals cNHEJ is the default pathway that repaired Cas9-generated breaks, whereas POLQ-mediated DSB repair only played a minor role. It is interesting to note that the loss of NHEJ pathway was fully compensated for by the activation of TMEJ, and, as a result, wt cells and NHEJ deficient cells have shown the equal efficiency of mutagenic DSB repair (Ferreira da Silva *et al.* 2019). The only effect of cNHEJ deficiency was a change in the repair signature showing that repair of Cas9-generated DNA breaks could efficiently be achieved in the absence of NHEJ. Compensation of the cNHEJ deficiencies by a higher contribution of TMEJ was also found in human HEK293T cell line and embryonic stem cells (van Overbeek *et al.* 2016; Schimmel *et al.* 2017). Finally, a comparison of our results with template-free SpCas9 mutagenesis in other organisms has shown that the DNA repair mutation profiles are composed of different contributions from the cNHEJ and TMEJ pathways, depending on cell types and species. A better understanding of the impact of cNHEJ and TMEJ in repair CRISPR/Cas-induced genomic DSB is expected to help to clarify DSB repair regulation and choose the optimal conditions for gene editing. The mutation outcome of template-free repair of CRISPR/Cas9-induced DSB found in our study should be taken into account when performing site-specific mutagenesis in *Chlamydomonas*.

### TMEJ is responsible for random integration of the transgenes co-transformed with donor ssOligos

One of the methods for the editing of non-selectable gene in *Chlamydomonas* is co-transformation of cells with SpCas9/gRNA RNP, a gene-specific DNA template, and a selective plasmid. This would be followed by PCR-screening for mutations in isolated colonies (Greiner *et al.* 2017). In this study we tested the effect of KU80, KU70, and POLQ deficiency on the random integration of pPmR in co-transformations with *SNRK2.2* specific SpCas9 RNP and ssODNs. Our data show that the deletion of the *KU80* or *KU70* genes did not change the rate of transgene integration in the genome of *Chlamydomonas* (Figure 4A). In contrast, the inactivation of the *POLQ* gene results in a significant decrease in the transformation efficiency, which is indicative of the dominant role of the TMEJ pathway for random transgene insertions into the nuclear genome (Figure 4). The prevention of random integration for our *polQ* mutants is consistent with the earlier study of plasmid transformation for *Chlamydomonas polQ* cells (Plecenikova *et al.* 2014). In this study, Plecenikova *et al.* demonstrated a 10-fold reduction in the transformation efficiency for the autotrophic *polQ* mutant compared with its parental strain, when using the plasmid vector pUCARG7.8, which complemented deficiency in ARG7 gene due to random insertion into the chromosome. This substantial role of the TMEJ pathway has also been found for mouse ES cells where the frequency of transfected DNA integration was not affected by the inactivation of proteins involved in cNHEJ. Instead, in POLQ deficient cells, it was reduced to 11% in comparison to the wt. This information demonstrates that POLQ was the main contributor for mediating the random integration of transgenes, whereas NHEJ has been observed to play a secondary role (Zelensky *et al.* 2017). These findings are similar to what we found for *Chlamydomonas*. However, in DT40 chicken cells, NHEJ has been reported to be responsible for almost all random transgene integration events (Iiizumi *et al.* 2008). In the human cell line Nalm-6, both NHEJ and TMEJ have been seen to be equal participants in random integrations (Iiizumi *et al.* 2008; Saito *et al.* 2017). Other studies have also revealed that mammalian cells deficient for both POLQ and NHEJ were devoid of random integration events (Saito *et al.* 2017; Zelensky *et al.* 2017). A proposed explanation for this result was improved site-specific knock-ins by HR (Jasin and Rothstein 2013). In the plant *Arabidopsis thaliana*, T-DNA integration also is believed to be critically dependent on POLQ. POLQ deficient mutant plants have been demonstrated to be resistant to T-DNA integration (van Kregten *et al.* 2016). Thus, the supporting literature and our data show that the contribution of cNHEJ and TMEJ to random integration of transgenes is dependent on the type of cell lines or species (Iiizumi *et al.* 2008; Saito *et al.* 2017; Zelensky *et al.* 2017). Knowledge about the impact of cNHEJ and TMEJ in random integration of supplied DNA could help to suppress undesirable insertion mutagenesis and increase the output of the CRISPR/Cas-mediated site-specific genome modifications. In *Chlamydomonas*, the dominant role of the TMEJ pathway is not only in random integration, but also is in CRISPR/Cas-mediated gene targeting. The important contribution of TMEJ in gene targeting impedes the use of *polQ* mutants as recipients to prevent random integration events for gene editing with ssODN repair templates.

### POLQ is responsible for most CRISPR/Cas-mediated gene targeting events

CRISPR/Cas-mediated HDR is a complicated process comprising three main sub-pathways in eukaryotic cells (Li *et al.* 2019). These sub-pathways that lead to precise genome editing without genomic destabilization include DSBR through the formation of Holliday junction structures, SDSA, and single-strand DNA incorporation ssDI (Kan *et al.* 2017). Target gene-specific dsDNA templates are used in the pathways described by the DSBR and SDSA models. In the ssDI pathway, the ssODN template is physically incorporated, unlike the SDSA pathway where the ssODN is used as a synthesis template (Kan *et al.* 2017; Boel *et al.* 2018; Kan and Hendrickson 2019). Single-stranded oligonucleotide templates are considered to be the most useful donors, because of their easier design and production, and less frequent random integration into the genome in comparison to similarly designed dsDNA templates (Würtele *et al.* 2003; Zorin *et al.* 2005). The major drawback of gene editing through SDSA pathway is the low rate of precision for editing cells, which is due to the multiple integrations of the exogenous DNA fragments into the target site (Paix *et al.* 2017; Boel *et al.* 2018). The most prominent reason for this complicated rearrangement is known to be the repeated template switching during the elongation stage (Boel *et al.* 2018). During this process, the elongating strand is able to withdraw from the first template and anneal to a microhomology in a second template to continue DNA synthesis before annealing back to the target. It has been suggested that various possible schemes for template switching during SDSA are based on microhomology and result in erroneous replication (Boel *et al.* 2018). Recently, template switching during CRISPR/Cas9-mediated HDR has been demonstrated in mammalian cells, zebrafish, and Atlantic salmon (Paix *et al.* 2017; Boel *et al.* 2018; Straume *et al*. 2020). HDR efficiency and rate of precise HDR events can be increased by optimization of homology arm length, symmetry of donor DNA templates, and template concentration (Renaud *et al.* 2016; Richardson *et al.* 2016; Boel *et al.* 2018; Yao *et al.* 2018; Straume *et al.* 2020). HDR efficiency using ssODN templates has been shown to be dependent on strand polarity (matched protospacer strand or spacer strand of a target; Ward 2015; Farboud *et al.* 2019). Farboud *et al.* (2019) demonstrated that the position of the modification relative to the PAM determined optimal oligo template polarity. It is important to note, that a frequency of target modification from ssODN decreased at a longer distance from the DSB (Ward 2015; Farboud *et al.* 2019). The effect of strand polarity can also be explained by slow and asymmetrical dissociation of SpCas9 from dsDNA substrates. Before complete dissociation, SpCas9 is known to release the 3′ end of the cleaved DNA strand, which was thought to be complementary to spacer strand repair template for the best for DSB repair (Richardson *et al.* 2016).

In *Chlamydomonas* transgenic cells, the CRISPR/Cas-mediated mut-*aphVIII* repair could occur via HR or a POLQ-mediated pathway that depends on a delivered repair template. We found that POLQ deficiency eliminated SpCas9-mediated HDR of the mut-*aphVIII* with single-stranded donor ODNs. Nevertheless, the mut-*aphVIII* could be restored by HR with long gene-specific dsDNA. We have detected a sharp decrease in the rate of SpCas9-mediated mut-*aphVIII* repair using the pHDR-APHVIII^Δ120^ in the *polQ* mutant when compared with the wt or *ku80* cells. These data show, that in the wt cells, TMEJ pathway could be a preferable method for the mut-*aphVIII* repair, even when using a repair substrate containing a long target-specific homology region suitable for HR. Recently it was performed the efficient SpCas9-mediated targeted insertions of long transgenic DNA fragments (1.7–6.4 kb) flanked only by short (25–50 bp) gene-specific homology arms or are without such arms which were not able to serve as substrates for HR (Kim *et al.* 2020; Picariello *et al.* 2020). In line with our data, we suggest an important role for POLQ in knock-ins directed by short homology in *Chlamydomonas* as well as in such knock-ins implemented in zebrafish, mammalian and *Drosophila* (Nakade *et al.* 2014; Hisano *et al.*, 2015; Kanca et al 2019; Wierson et al 2020).

### Disruption of the *KU80* gene resulted in stimulation of HDR/gene inactivation

Another strategy to increase HDR efficiency is based on the inhibition of the cNHEJ pathway, which competes with HDR (Ryu *et al.* 2019). Suppression of cNHEJ key proteins promotes the efficiency of HDR in *Drosophila*, *C. elegans* and mammals (Beumer *et al*. 2008; Böttcher *et al.* 2014; Chu *et al.* 2015; Ma *et al.* 2016; Maruyama *et al.* 2015; Ward 2015). In *Chlamydomonas ku80* strains, a higher rate of both the SpCas9-mediated mut-*aphVIII* repair and the SpCas9-mediated erroneous FLAG integration was found for single-stranded oligonucleotide templates that matched both the protospacer strand and the spacer strand of the target. Weak stimulation of mut-*aphVIII* repair was also detected for the donor plasmid pHDR-APHVIII^Δ120^ containing a long *APHVIII*-specific region. Complex FLAG insertion (shown in Supplementary Figure S7C) could be due to the template switching during SDSA pathway. Our data shows a similar range (2–6%) of the correctly integrated FLAG in wt and *ku80* cells (Figure 5B, highlighted in red). Stimulation of GT in the absence of KU70/KU80 could be associated with more efficient resection of DNA ends to produce 3′ overhangs on both sides of the DSB. These can then anneal to repair templates and prime DNA synthesis during the SDSA pathway.

Our study has found that both cNHEJ and TMEJ are involved in template-free repair of SpCas9 generated DSB. We have also demonstrated that POLQ deficiency severely impeded random integration of transgenes. These findings suggest a secondary role of NHEJ in this process. POLQ could be the main contributor to mediating CRISPR/Cas-induced HDR and mediate random integration of transgenes. Linear DNA fragments, including short gene-specific homology arms, could be are the most promising donors for CRISPR/Cas-mediated gene editing in *Chlamydomonas*. Our data also show that the disruption of the *KU80* gene results in significant stimulation of homologous targeted gene repair/inactivation using donor ssODN templates. This is potentially due to an activation of the TMEJ pathway and could help in the development of new recipient strains for algal biotechnology. Our DNA repair-deficient strains used are available to the scientific community and are expect to be useful for further recombination studies in *Chlamydomonas*. In summary, our study thus opens new perspectives for sustainable algal biotechnology avenues.
